# Development of Accident Probability Index Using Surrogate Indicators of Chemical Accidents in Chemical Plants

**DOI:** 10.3390/ijerph16183271

**Published:** 2019-09-05

**Authors:** Da-An Huh, Eun-Hae Huh, Sang-Hoon Byeon, Jong-Ryeul Sohn, Kyong Whan Moon

**Affiliations:** 1 Department of Health Science, Korea University, Anam-ro 145, Seongbuk-gu, Seoul 02841, Korea (D.-A.H.) (E.-H.H.) (J.-R.S.); 2 Department of Health and Safety Convergence Science, Korea University, Anam-ro 145, Seongbuk-gu, Seoul 02841, Korea

**Keywords:** accident probability index, chemical accident, risk index, surrogate indicators

## Abstract

To reduce damage caused by chemical accidents, it is important to establish a prevention system for chemical accidents. The first step in the prevention of chemical accidents is to screen the high-risk chemical plants. Risk index, one of the screening methods, can indirectly estimate the risk at each chemical plant. For calculating the risk index, the probability of an accident needs to be estimated, which requires complex calculation and confidential data from plants that are difficult to obtain. Therefore, we developed a new index, the accident probability index, to estimate accident probability in chemical plants using readily accessible data. We conducted a literature survey on the existing risk indices and interviewed chemical experts and government chemical managers to select surrogate indicators related to chemical accidents, and four indicators were chosen: hazardous characteristics of chemicals, handling volume, records of accident frequency, and national accident frequency of chemicals. We calculated the accident probability index for 4520 chemical plants, and index value means was 5.324 (95% confidence interval (CI): 3.156, 7.493). An increase by 10 in the index value denoted a 1.06-fold (95% CI: 1.04, 1.08) increase in the odds ratio for actual accident occurrence. The accident frequency of the fourth quartile of the index value was 4.30 times (95% CI: 1.72, 10.75) higher than those of the first quartile.

## 1. Introduction

The frequency of chemical accidents has been increasing with the development of the industry and increments of various chemicals [1]. Since the accident of hydrofluoric acid that occurred in 2012 at Gumi Industrial Complex in Korea, public concern and needs for management of hazardous chemical accidents have increased. The Korean Ministry of Environment has established a new chemical management law called Korea Chemicals Control Act to respond quickly to chemical accidents [2].

To reduce damage from chemical accidents, a prevention system for chemical accidents needs to be formed while also establishing a rapid response system. The first step in the prevention system is to screen the chemical plants that are at a high risk. The risk index, which is one of the screening methods, can indirectly estimate the risk at each chemical plant [3]. The government can select chemical plants with a high-risk index as priority targets for management.

However, the existing risk indices for screening of the high-risk chemical plants have some limitations. The main factor for calculating the risk indices involves estimating the probability of an accident at a chemical plant. These estimation methods, however, involve complex calculations and require confidential data from each plant, which are hard to access [3]. Therefore, for managing chemical plant accidents at the national level using the risk index, a new method is needed for the government to estimate the probability of an accident using readily available data.

The aim of this study is to propose a new index called the accident probability index that can estimate accident probability in chemical plants using surrogate indicators. The term ‘surrogate indicators’ denotes readily accessible indicators that can be used to replace the inaccessible indicators related to the frequency of chemical accidents. In this study, we present the process of selecting the surrogate indicators, the equation for calculating the accident probability index, and the results of the reliability test of the index. We expect that our results could be applied for the national management of accidents in chemical plants.

## 2. Materials and Methods

### 2.1. Selection of the Key Indicators

**Figure 1:**
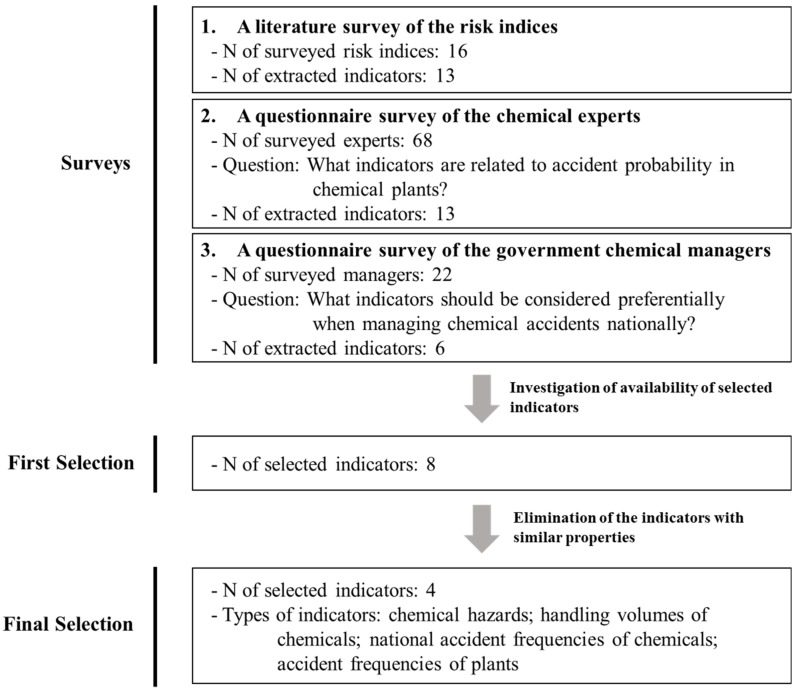
The flow chart of the process of selecting surrogate indicators.

To simplify the estimation process of accident probability, we needed to minimize the number of indicators used in the equation for calculating the accident probability index. Therefore, we selected key indicators related to accident probability using three methods and designed the index equation using these indicators. First, we conducted literature surveys to investigate the types of indicators used to estimate accident probability in the existing risk indices. Sixteen existing indices were investigated, and we extracted 13 indicators used to predict the accident frequency of plants in each index. The risk indices we surveyed are as follows: safety risk index; environmental hazard index (EHI); Dow fire and explosion index (Dow F & EI); transportation risk indices of chemicals; Mond fire, explosion, and toxicity index (Mond FETI); environment-accident index (EAI); hazard identification and ranking (HIRA); accident hazard index; risk index Z; hazardous waste index (HWI); environmental risk index (ERI); toxicity hazard index (THI); safety-weighted hazard index (SWeHI); inherent safety index (ISI); integrated inherent safety index (I2SI); and risk-screening environmental indicators (RSEI) [4,5,6,7,8,9,10,11,12,13,14,15,16,17,18,19]. Second, we interviewed chemical experts with chemical management experience of at least 10 years. The experts answered our question, “What indicators are related to accident probability in chemical plants?”. A total of 68 experts participated in this interview, and we extracted 13 indicators from this result. Third, we conducted interviews with government chemical managers. The managers answered our question, “What indicators should be considered preferentially when managing chemical accidents nationally?”. A total of 22 managers participated in this interview, and we extracted 6 indicators from this result. Of the indicators extracted from the three surveys, we selected 8 indicators based on data availability, of which we excluded 4 indicators with similar properties (Figure 1). Finally, the following 4 indicators were selected as surrogate indicators for calculating the accident probability index: hazardous characteristics of chemicals, handling volume, national accident frequencies of chemicals, and records of accident frequencies of plants. Table 1 presents the indicators extracted from each survey.

### 2.2. Design of a Basic Form of the Equation

The selected indicators were divided into two categories: information about chemical plants and information about chemicals. We designed the equation so that the plant using many chemicals has a large index because various chemicals can be used at the same time in each plant. Therefore, a basic form of the index equation is given as:

(1) $$\mathrm{Accident}\;\mathrm{probability}\;\mathrm{index}\;=\;F\left(f\right)\times \sum_{i=1}^{n}\left(A_{i}\times B_{i}\times C_{i}\right)$$

where F is the standardizing function, f is the accident frequency of each plant, n is the number of chemicals used in each plant, $A_{i}$ is the health hazard value of chemicals used in each plant, $B_{i}$ is the handling volume of chemicals in each plant, and $C_{i}$ is the national accident frequency of chemicals.

### 2.3. Sources of Data

Hazardous information on chemicals was obtained from National Institute of Technology and Evaluation (NITE). NITE provides hazardous information on 3967 chemicals classified according to the Globally Harmonized System (GHS). We used 3028 out of 3967 chemicals after excluding those that did not have a CAS number and those that had overlapping chemicals. In case of the data on chemical plants, the Ministry of Environment and Han River Regional Environmental Office in Korea has provided information on 4520 chemical plants located in Seoul and Gyeonggi-do regarding the records of accidents in plants, types of chemicals used in each plant, and annual handling volume of chemicals during 2003–2016. Although these chemical plants are limited in the metropolitan area, they are involved in various chemical industries and are not biased to a specific industry. We added the accident history information provided by the Chemistry Safety Clearing-house (CSC, https://csc.me.go.kr) for 2014–2018 to these data. We considered the number of accidents of the plant which did not have the accident records in 2003–2018 as zero. The national accident history of chemicals was also collected by the CSC.

### 2.4. The Calculation of the Comprehensive Hazardous of Chemicals by Using Mahalanobis–Taguchi System

To quantify the comprehensive hazards of chemicals, we used the Mahalanobis–Taguchi System (MTS), which is a chemical ranking and scoring (CRS) method. MTS presents various variables related to the hazards of chemicals as a simple indicator, Mahalanobis distance (MD) [20,21,22,23]. The first step to calculate MDs is constructing a Mahalanobis space (MS) which is used as the reference group. When we evaluate the harmful effects of chemicals, MS could be constructed with chemicals that are relatively less harmful. Because MD is calculated by the distance from the center of the MS, a larger MD indicates that chemicals are more hazardous. In addition, MTS can estimate the relative chemical hazards more accurately than the existing CRS methods because it considers the correlation between hazardous variables [24,25].

We used the following 10 variables of GHS to calculate the MD: acute toxicity (oral), acute toxicity (dermal), acute toxicity (inhalation), skin corrosion/irritation, serious eye damage/eye irritation, germ cell mutagenicity, carcinogenicity, reproductive toxicity, specific target organ toxicity (single exposure), and specific target organ toxicity (repeated exposure). According to the GHS classification criteria, each variable is represented from Category 1 to Category 5, and Category 1 indicates the highest hazard. If the hazard of a chemical is outside the classification criteria, it is categorized as Not Classified, and if a chemical is outside the GHS definition, it is categorized as Not Applicable. If a chemical does not have enough hazard information, it is categorized as Classification Not Possible [26]. We assigned 100 points to Category 1, the most dangerous classification of each variable, and a smaller score to a less hazardous category. We allocated 10 points to the Not Classified category which is lower than Category 5 and 1 point to the Not Applicable category which is the lowest score. In the case of Classification Not Possible, we allocated 30 points close to the score in Category 3 because the absence of information does not mean there is no hazard. Table 2 presents the quantification results of health hazard levels.

Another step for calculating MD is constructing a unit space, called the MS, which is used as a reference group. Because MS should consist of characteristics of relatively low-hazard chemicals, we selected the reference chemicals following two steps. First, we excluded chemicals that had at least one Category 1 or Category 2 classification that indicates a high level of hazard. Second, chemicals with a total score of 280 or more for the 10 health hazard variables were excluded. For example, formaldehyde (CAS No. 50-00-0) was excluded in the reference group because of Category 1 in carcinogenicity, and 1-heptanol (CAS No. 111-70-6) was also excluded because of Category 2 in skin corrosion/irritation. In contrast, we considered flutolanil (CAS No. 66332-96-5) as a relatively low-hazard chemical because it did not have Category 1 and Category 2, and it had a total score of 120 which is lower than 280. According to this standard, we selected 151 chemicals as reference chemicals.

### 2.5. Standardization of Indicators

The selected indicators were required to be standardized because they have different ranges and units. Therefore, we divided each indicator into 5 classes using Jenks natural breaks classification method, which is a data clustering method designed to determine the best arrangement of values into different classes. Jenks natural breaks classification is performed by seeking to minimize each class’s average deviation from the class mean while maximizing each class’s deviation from the means of other groups [27,28]. We assigned scores from 1 to 5 in five classes and used these scores in the equation for calculating the accident probability index.

In the case of the accident history of a plant, the range of the indicator is not wide enough to use Jenks natural breaks classification because chemical accidents are a rare event. Therefore, we used the logistic function to standardize the accident history of the plant and modified the function so that it could have a range from 1 to 5 points. The modified function is:

(2) $$F\left(f\right)=\;\frac{K}{1+e^{-\left(f-a\right)}}+b\;(\mathrm{range}:\;1\;\mathrm{to}\;5)$$

where $F\left(f\right)$ is the standardized score, f is the accident frequency of each plant, and K, a, and b are constants.

### 2.6. Reliability Test of the Accident Probability Index

To test the reliability of the index, we analyzed the statistical association between the index and the actual occurrence of chemical accidents. However, we could expect a high correlation between these two values because the number of accidents at the plant was used as an indicator to calculate the accident probability index. Therefore, we needed to divide the accident history of plants into two groups. In the 2003–2018 accident history collected in this study, we used data from 2003–2015 to calculate the index and data from 2016–2018 to validate the index. A logistic regression analysis was performed to analyze the association between the index and the actual occurrence of chemical accidents. The dependent variable is a dichotomous variable that indicates the occurrence of accidents for 2016–2018 years (0: no accident, 1: at least one accident occurred). All statistical analyses used for reliability tests were conducted using SPSS version 25.0 (IBM, New York, NY, USA).

## 3. Results

**Figure 2:**
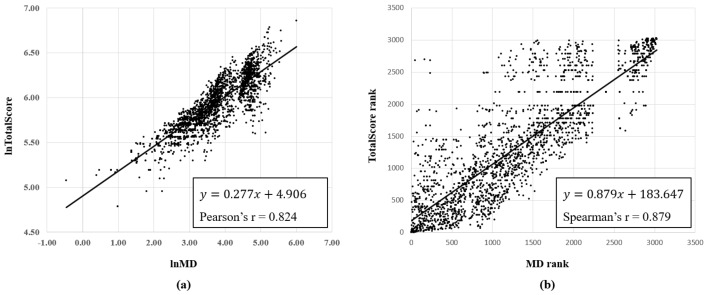
Results of correlation analysis between Mahalanobis distance (MD) and total score using Globally Harmonized System (GHS) categories: (**a**) Pearson correlation analysis and (**b**) Spearman rank correlation analysis.

Figure 2 shows the results of correlations between MD and the total score using GHS categories of 3028 chemicals. The Pearson correlation coefficient for the total score and MD was 0.824 (*p* < 0.001), and a high positive correlation was observed. If the Spearman rank correlation analysis was conducted by changing the total score and MD to the rank of chemicals, the Spearman correlation coefficient was 0.879 (*p* < 0.001), and a high positive correlation was also observed. However, the rankings using total scores and MD for 3028 chemicals did not perfectly match.

Under the Chemicals Control Act in Korea, hazardous chemicals are classified into the following four types according to their hazard properties: chemicals requiring preparation for accidents, toxic chemicals, prohibited chemicals, and restricted chemicals. We investigated the distribution of MD according to the type of chemical to investigate whether the MD properly reflected the hazards of the chemicals. Table 3 shows the means and quartiles of the MD according to the types of chemicals. The mean of MD for the total chemicals was 54.67, and a distribution skewed to the left was observed. The mean of MD for the reference chemicals composing the MS was the lowest at 1.20, and the averages of MD for chemicals requiring preparation for accidents and toxic chemicals were 100.97 and 79.95, respectively. The means of MD for prohibited chemicals and restricted chemicals were 14.35, 86.99, respectively, while the average of MD for other chemicals not included in the four types was 42.65.

**Figure 3:**
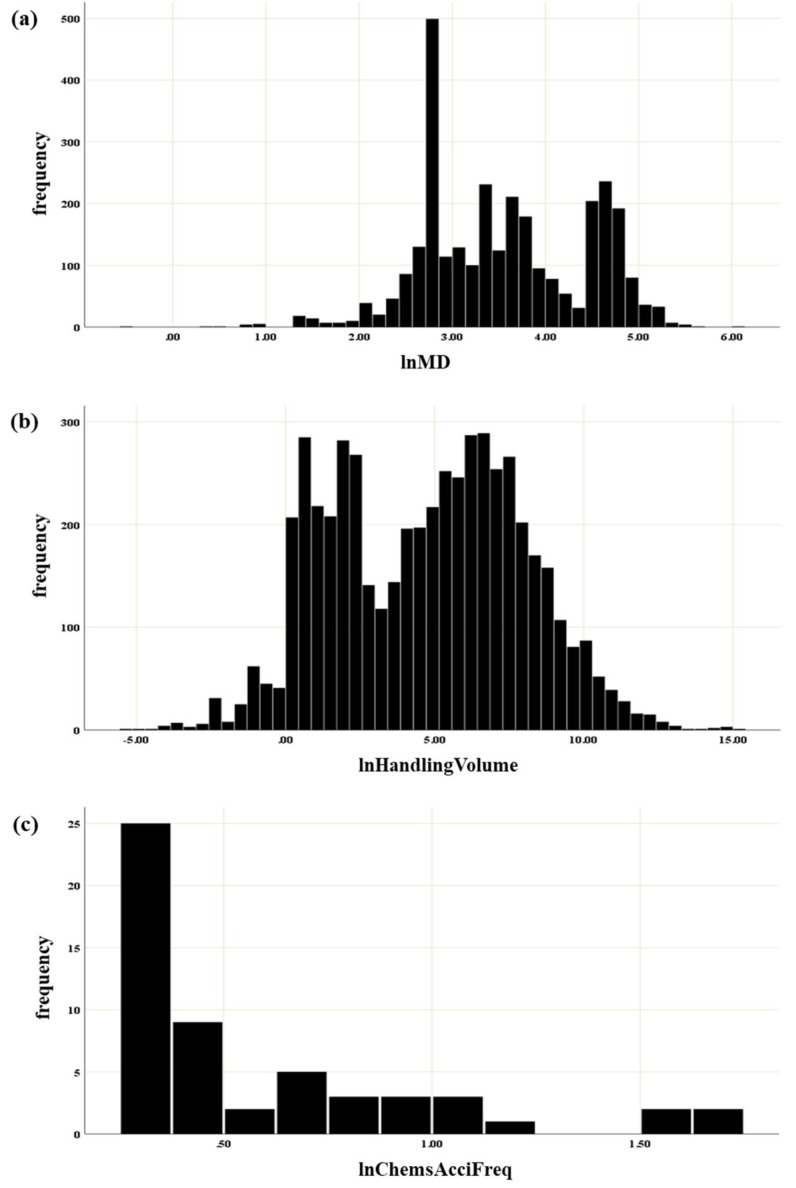
Distribution of the three surrogate indicators: (**a**) Mahalanobis distance of chemicals, (**b**) handling volume of chemicals, and (**c**) national accident frequency of chemicals.

Figure 3 presents the distribution of three indicators, MD, handling volume, and national accident frequency of chemicals. Each distribution was divided into five classes by Jenks natural breaks classification; Table 4 shows the classification criteria for each indicator. The first level was defined as 3, 5 tons, and 1 time or less, respectively, and one point was allocated. Similarly, the fifth level was defined as above 100, 2000 tons, and 15 times, respectively, and five points were allocated.

**Figure 4:**
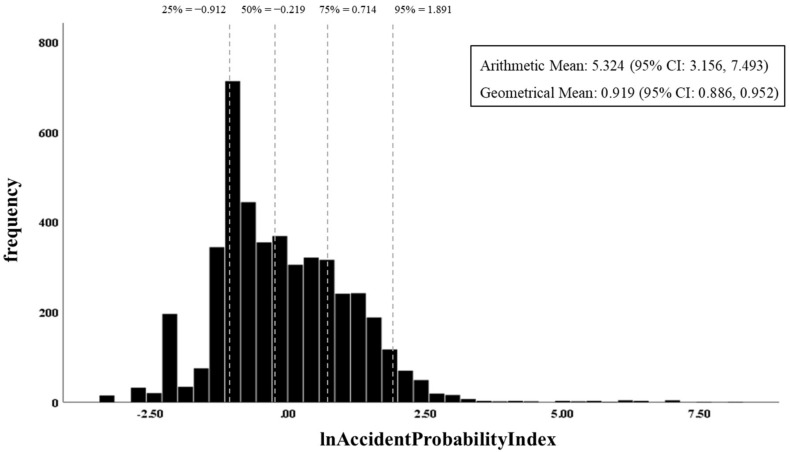
Distribution of accident probability index for 4520 chemical plants.

Figure 4 shows a histogram of the accident probability index calculated by using the results of equation 1, 2, and Table 4. The index showed lognormal distribution skewed to the left. The range of index was 0.033 to 3697.282, and the arithmetic mean of the index was 5.324 (95% confidence interval (CI): 3.156, 7.493) and the geometrical mean of the index was 0.919 (95% CI: 0.886, 0.952). The 95th percentile value of the accident probability index was 6.626 (=$e^{1.891}$), which shows that the arithmetic mean is significantly affected by some extreme index values.

Table 5 presents the odds ratio (OR) of the accident probability index for the actual occurrence of chemical accidents. In the case of considering the index as continuous, 10 increments of the accident probability index were associated with a 1.06-fold (95% CI: 1.04, 1.08) increase in the OR for the actual chemical accident. The OR for accident occurrence in plants with the fourth quartile of the index was 4.30 times (95% CI: 1.72, 10.75) higher than the plants with the first quartile. In addition, OR for accident occurrence tended to increase with the increment of quartile (*p* for trend <0.001).

## 4. Discussion

In this study, we developed an accident probability index that can estimate accident probability in chemical plants using surrogate indicators that are relatively easy to access information. According to the literature surveys and interviews with chemical experts and chemical managers of government, four indicators (chemical health hazards, handling volume of chemicals, national accident frequency of chemicals, and accident frequency of plants) were selected, and we calculated the index using these indicators. We compared the index with actual accident occurrences and found that 10 increments of accident probability index were associated with a 1.06-fold increase in the OR for the actual chemical accident.

We extracted various indicators related to chemical accidents through literature surveys on existing risk indices and interviews with experts and managers of chemical industries. The indicators chosen by experts were mostly similar to those used in existing risk indices, whereas those chosen by government chemical managers were different. The managers agreed that information on plant processes and safety management is needed to estimate the accident probability of plants but commented that these data are difficult to obtain nationally. The experts recommended using surrogate indicators that represent the physical hazard of chemicals and safety facilities in the plant, if data are not available because these properties are key factors for estimating the accident probability [3,6,7,9,17,18,29]. We assumed the following two conditions to select the surrogate indicators: chemicals with high physical hazard tend to lead to chemical accidents more frequently, and plants with insufficient safety facilities have had frequent chemical accidents. As a result of the discussion, the physical hazard of the chemical was replaced by the national accident frequency of chemicals, and the status of safety facilities in the plants was replaced by records of accident frequency of the plant.

The main property of the accident probability index presented in this study was that the comprehensive hazards of chemicals were assessed through the new CRS method, MD. Although there are various CRS methods [30,31,32,33,34,35], MD differs because it considers the correlation of variables that have similar characteristics. According to the results presented in Figure 2, MD and the total score using GHS have a high correlation because the MD is calculated using GHS scores. However, the rankings of chemicals using MD and GHS scores are not perfectly consistent because the MD presents results considering the interactions between variables. The health hazard variables are not independent, and some variables may affect each other [36]. For example, Demeton (CAS No. 8065-48-3) used as an insecticide has a GHS score of 710 and the 20th highest score among 3028 chemicals. This chemical was assigned 100 points, which is equivalent to Category 1, with acute toxicity (oral) and acute toxicity (dermal), but these two variables are not independent and share about 60% hazard characteristics [37]. Therefore, the total score using GHS without considering the correlation between variables may overestimate the actual hazards of this chemical. In the case of using MD, the correlation between the two variables was adjusted, and the chemical Demeton was ranked 96th out of 3028 chemicals, which is lower than the ranking using the GHS score.

Another strength of MD is that MDs reflect the characteristics of chemicals requiring preparation for accidents. Under the Korean Chemicals Control Act, chemicals with high probability of accidents and high health hazard are classified as chemicals requiring preparation for accidents [2]. According to the results presented in Table 3, chemicals requiring preparation for accidents had the highest average of MD among all types of chemicals. This indicates that we could screen chemicals that have a high accident probability and health hazard by using the MD. Therefore, we believe that using MD in the accident probability index to estimate accident probability in plants is the most appropriate method of CRS.

The accident probability index calculated using four surrogate indicators is highly associated with the actual occurrence of chemical accidents. According to the results presented in Table 5, the increase of index in the plant by 10 resulted in a 1.06-fold increase in the OR for chemical accidents. In addition, the plants corresponding to the fourth quartile of the index had 4.30 times higher OR for chemical accident than the plants corresponding to the first quartile. These trends were observed in the year 2019. According to media reports, chemical accidents occurred at seven plants from January to June 2019 in Korea. Of these seven chemical plants, the accident probabilities in only three plants could be calculated by the accident probability index using data provided by government agencies. The indices of three plants where chemical accidents occurred were 1.606, 1.841, and 159.968 respectively, and interestingly, all three plants corresponded to the third or fourth quartile of the index. These results indicate that the accident probability index presented in this study can reliably estimate the accident probability in plants.

The accident probability index has several strengths. First, the chemicals used in the index were ranked using MDs which could assess the comprehensive hazard of chemicals more accurately than other CRS methods. According to a previous study, MD reflects the overall hazard of chemicals better than the existing CRS methods, European Union Risk Ranking Method (EURAM) and Chemical Hazard Evaluation for Management Strategies (CHEMS) [37,38,39]. Second, the accident probability index is calculated from relatively readily accessible data. Therefore, chemical managers in the government can select which plants need to be managed first, even if detailed information on each plant is not available. If the managers use the geographic information system, they can screen the areas that need to be controlled more quickl y. Third, the accuracy of the index can be improved continuously by obtaining additional information. According to the results presented in Table 1, many variables could not be used for index calculation because the government was not able to access these data. This result provides directions for what additional data the government needs to obtain, and we can expect an improvement in the accuracy of the index by using this additional information.

The accident probability index also has a limitation. Although the index for each plant is calculated, it does not represent the exact accident probability of the plant. The index is a relative value and can only be used for relative comparisons to assess which plant has a higher accident probability.

## 5. Conclusions

In this study, we presented an accident probability index to evaluate the accident probability in chemical plants by using readily accessible surrogate indicators. The increase in the index was related to an increase in OR for the actual occurrence of chemical accidents. These results indicated that the new index could evaluate the accident probability of each chemical plant more easily, and we expect this index to be applied to risk indices for efficient national management of chemical plants.

## Figures and Tables

**Table 1:** Indicators extracted through a literature survey and interview with chemical experts and government chemical managers.

Information Types	Literature Survey	Chemical Experts	Government Chemical Managers	Availability	Used Variable
Material	hazardous characteristics of chemicals	hazardous characteristics of chemicals	hazardous characteristics of chemicals	○	○
	handling volume	handling volume	handling volume	○	○
	heat of reaction	-	-	-	-
	distribution of stored chemicals	-	-	-	-
	-	-	storage volume	○	-
	-	-	legal management status of chemicals	○	-
	-	-	number of handling chemicals	○	-
Process in plant	type of process	type of process	-	-	-
	deterioration status of equipment	deterioration status of equipment	-	-	-
	number of major processes in plants	-	-	-	-
	temperature and pressure of process	temperature and pressure of process	-	-	-
	-	complexity or density of plant	-	-	-
Accident frequency	records of accident frequency	records of accident frequency	records of accident frequency	○	○
	-	national accident frequency of chemicals	-	○	○
Management	protection process	protection process	-	-	-
	management or emergency plan	management or emergency plan	-	-	-
	safety distance	-	-	-	-
	human error	human error	-	-	-
Miscellaneous	-	meteorological data	-	○	-
	-	training for the worker	-	-	-

○: xxx.

**Table 2:** Results of quantification of health hazard variables.

Categories	Category 1	Category 2	Category 3	Category 4	Category 5	Not Classified	Not Applicable	Classification Not Possible
Score	$\frac{100}{1}=100$	$\frac{100}{2}=50$	$\frac{100}{3}=33.3$	$\frac{100}{4}=25$	$\frac{100}{5}=20$	10	1	30

**Table 3:** Arithmetic mean, 95% confidence interval, and quartiles of Mahalanobis distance by the types of chemicals classified according to the Korea Chemicals Control Act.

Chemical Types	*N*	Mahalanobis Distance			
		Mean (95% CI ^1^)	Q1 ^2^	Q2 ^3^	Q3 ^4^
Total	3028	54.67 (53.11, 56.23)	19.74	36.93	92.38
Reference	151	1.20 (1.02, 1.37)	0.56	1.01	1.70
CRPA ^5^	97	100.97 (90.36, 111.57)	56.51	107.31	126.97
TC ^6^	647	79.95 (69.11, 90.78)	35.71	81.12	105.14
RC ^7^	40	86.99 (67.98, 105.99)	34.87	85.27	131.49
Other chemicals	2315	42.65 (41.09, 44.22)	15.74	27.39	51.44

^1^ CI: confidence interval. ^2^ Q1: first quartile. ^3^ Q2: second quartile. ^4^ Q3: third quartile. ^5^ CRPA: chemicals requiring preparation for accidents. ^6^ TC: toxic chemicals. ^7^ RC: restricted chemicals.

**Table 4:** Classification criteria and quantification results of each indicator.

Indicators	Level 1	Level 2	Level 3	Level 4	Level 5
Mahalanobis distance (A)	≤3	≤15	≤25	≤100	100<
Handling volume (ton) (B)	≤5	≤50	≤400	≤2000	2000<
National accident frequency of chemicals (C)	≤1	≤3	≤10	≤15	15<
Score	1.0	2.0	3.0	4.0	5.0

**Table 5:** Results of logistic regression analysis for actual occurrence of chemical accidents in 2015–2018 by the accident probability index.

Indicator	*N* of Plants with Accidents /*N* of Total Plants	%	Odds Ratio (95% CI ^1^)
Per 10 increasing of accident probability index			1.06 (1.04, 1.08)
Index value quartile (range)			
Q1 (0.034–0.402)	6/1429	0.42	Ref.
Q2 (0.435–0.803)	3/960	0.31	0.74 (0.19, 2.98)
Q3 (0.837–2.041)	9/1009	0.89	2.13 (0.76, 6.02)
Q4 (2.075–3697.282)	20/1122	1.78	4.30 (1.72, 10.75)
*p* for trend			<0.001

^1^ CI: confidence interval.
